# Nanomotion technology: an innovative method to study cell metabolism in Escherichia coli, as a potential indicator for tolerance

**DOI:** 10.1099/jmm.0.001912

**Published:** 2024-11-08

**Authors:** Christèle Aubry, Carole Kebbi-Beghdadi, Amanda Luraschi-Eggemann, Gino Cathomen, Danuta Cichocka, Alexander Sturm, Gilbert Greub, The ERADIAMR Consortium§

**Affiliations:** 1 Institute of Microbiology, Lausanne University Hospital and Lausanne University, CH-1011 Lausanne, Switzerland; 2 Resistell AG, Hofackerstrasse 40, CH-4132 Muttenz, Switzerland

**Keywords:** antibiotic, growth-independent method, nanomotion, rapid AST, tolerance

## Abstract

**Introduction.** Antibiotic tolerance corresponds to the bacterial ability to survive a transient exposure to antibiotics and is often associated with treatment failure. Current methods of identifying tolerance based on bacterial growth are time-consuming. This study explores the use of a growth-independent method utilizing nanomotion technology to detect antibiotic-tolerant bacteria.

**Hypothesis.** The nanomotion signal obtained from a nanomechanical sensor measures real-time metabolic activity and cellular processes and could provide valuable information about the tolerance of bacteria to antibiotics that cannot be detected by standard antibiotic susceptibility tests.

**Aim.** The aim of this study is to investigate the potential of nanomotion technology to record antibiotic-tolerant bacteria.

**Methodology.** We generated a slow-growing *Escherichia coli* strain by manipulating *mazF* expression levels and confirmed its viability by several standard methods. We subsequently measured its nanomotion and the nanomotion of the WT *E. coli* in the presence or absence of antibiotics. Supervised machine learning was employed to distinguish slow-growing from exponentially growing bacteria. Observations for bacterial nanomotions were confirmed by standard kill curves.

**Results.** We distinguished slow-growing from exponentially growing bacteria using specific features from the nanomotion signal. Furthermore, the exposition of both growth phenotypes to polymyxin decreased the nanomotion signal indicating cell death. Similarly, when exponentially growing cells were exposed to ampicillin, an antibiotic whose efficacy depends on the growth rate, the nanomotion signal also decreased. In contrast, the nanomotion signal remained unchanged for slow-growing bacteria upon exposure to ampicillin. In addition, antibiotic exposure can cause bacterial elongation, in which the biomass of a cell increases without cell division. By overexpressing *sulA*, we mimicked antibiotic-induced elongation. Differences in the nanomotion signal were observed when comparing elongating and non-elongating phenotypes.

**Conclusion.** This work shows that nanomotion signals entail information about the reaction to antibiotics that standard MIC-based antibiotic susceptibility tests cannot detect. In the future, nanomotion-based antibiotic tolerance tests could be developed for clinical use in chronic or relapsing infections.

## Introduction

Antibiotic treatment failure is a global health issue typically accredited to bacterial resistance. Resistance mechanisms are diverse and are due to mutations or the acquisition of resistance genes by bacterial foreign DNA, such as plasmids [1]. These mechanisms decrease the effectiveness of antimicrobial agents, increasing the MIC of resistant strains compared with susceptible strains. The measurement of MICs is the standard in diagnostic laboratories to determine bacterial susceptibility to antibiotics. MIC corresponds to the lowest concentration at which no growth is detected [2]. It is generally performed in liquid or solid media by exposing bacteria to a gradient of antibiotic concentrations during 16–20 h. Such tests are easy and often automated and miniaturized using microfluidics and are considered the ‘gold standard’ for susceptibility testing. However, they withhold information about antibiotic tolerance [3], another mechanism that can lead to treatment failure. Although its impact on infections on promoting the evolution of antibiotic resistance is known, tolerance is rarely considered in the healthcare setting [4], mainly due to the lack of rapid, efficient and robust testing approach. Many relapses are due to antibiotic-tolerant bacteria being unharmed by antibiotic exposure [5,6] – a phenomenon observed in acute and chronic infections, including endocarditis, urinary tract infection, cystic fibrosis and *Mycobacterium tuberculosis* infections.

Tolerance refers to the ability of a bacterial population to survive a transient exposure to bactericidal antibiotics at concentrations otherwise toxic for non-resistant strains [3,7,10]. In most cases, tolerance occurs during reduced metabolic activity in non-growing or slow-growing bacteria [11,12]. Many mechanisms can induce a state of bacterial tolerance, such as genetic mutations, exposure to inhibitors, location within a biofilm or starvation [11,13]. Bacteria in such a state survive bactericidal antibiotics whose killing efficacy requires active growth, e.g. β-lactams [11,14,17]. This extensive class of drugs inhibits cell wall synthesis by preventing the formation of peptidoglycan bonds [18]. Defects in the bacterial peptidoglycan layer are predominantly introduced during growth and cell division. Thus, a strong correlation between tolerance and slow growth was demonstrated in *Escherichia coli*, where the killing rate by β-lactams is proportional to the rate of bacterial growth [14]. Comparable results with other antimicrobial agents, such as fluoroquinolones, have also been observed [19]. Consequently, MIC measurements (performed under standard growth conditions) do not allow differentiating tolerant from non-tolerant bacteria.

Laborious time-kill curves based on the measurement of c.f.u. over time [20] can reveal antibiotic tolerance and are the standard method. In such cases, prolonged exposure to an antimicrobial agent is needed to produce the same level of killing as for a non-tolerant strain. To quantify tolerance, the minimum duration of killing (MDK), an extracted value from the time-kill curves, has been proposed [11]. MDK is the time needed to kill a given proportion of the population at an antibiotic concentration higher than MIC. For example, if a strain has a higher MDK than the WT, it demonstrates that the bacterial killing requires more time, which corresponds to a higher antibiotic tolerance [20].

Because of being labour-intensive and time-consuming, we were interested in exploring methods besides kill curves to identify tolerant bacteria. Through the past 10 years, cantilevers (or nanomechanical sensors) based on atomic force microscopy (AFM) have been introduced to aid versatile and rapid tests to determine antibiotic resistance [21]. Initially, AFM was described as a revolutionary microscope able to investigate sample surfaces on an atomic scale and reconstruct the 3D topography of a specimen [22]. Vice versa, molecules or living cells can also be attached to cantilevers. The Phenotech device using such a technology records natural nanomotions of living organisms and mass variations due to chemical treatments or cell division [22,25]. In addition, oscillations of cantilevers holding bacteria were reduced under conditions of antibiotic exposure compared with those with untreated bacteria [24]. Ever since, this method has been applied to evaluate antibiotic susceptibility profiles of several bacteria such as *E. coli* [24,26,28], *Bordetella pertussis* [29] and *Staphylococcus aureus* [30] and even slow-growing bacteria such as *Mycobacterium abscessus* [31].

The objectives of this study were to (i) determine if the nanomotion approach is suitable to differentiate tolerant from non-tolerant phenotypes and (ii) study the impact of bacterial elongation (increased mass per bacterial cell) on the nanomotion signal. Therefore, we assessed the difference between the nanomotion signals of an exponentially growing WT *E. coli* strain (carrying the empty plasmid pBAD101), a slow-growing strain (pBAD101-*mazF*) and a growing but non-dividing (i.e. elongating) strain (pBAD101-*sulA*). The *mazF-*expressing strain was used in this work as a surrogate of the tolerant phenotype. The *sulA-*expressing strain was used as a surrogate of elongated bacteria.

## Methods

### Bacterial strains and growth conditions

All strains and plasmids used are listed in Table 1.

**Table 1.** Bacterial strains and plasmids used for this study

Strain	Plasmids	Relevant characteristics
*E. coli* MG1655	pBAD101	Exponential-like WT growth
*E. coli* MG1655	pBAD101-*mazF*	Slow growth with or without arabinose induction
*E. coli* MG1655	pBAD101-*sulA*	Growing but non-dividing cells upon arabinose induction

**Fig. 1.**
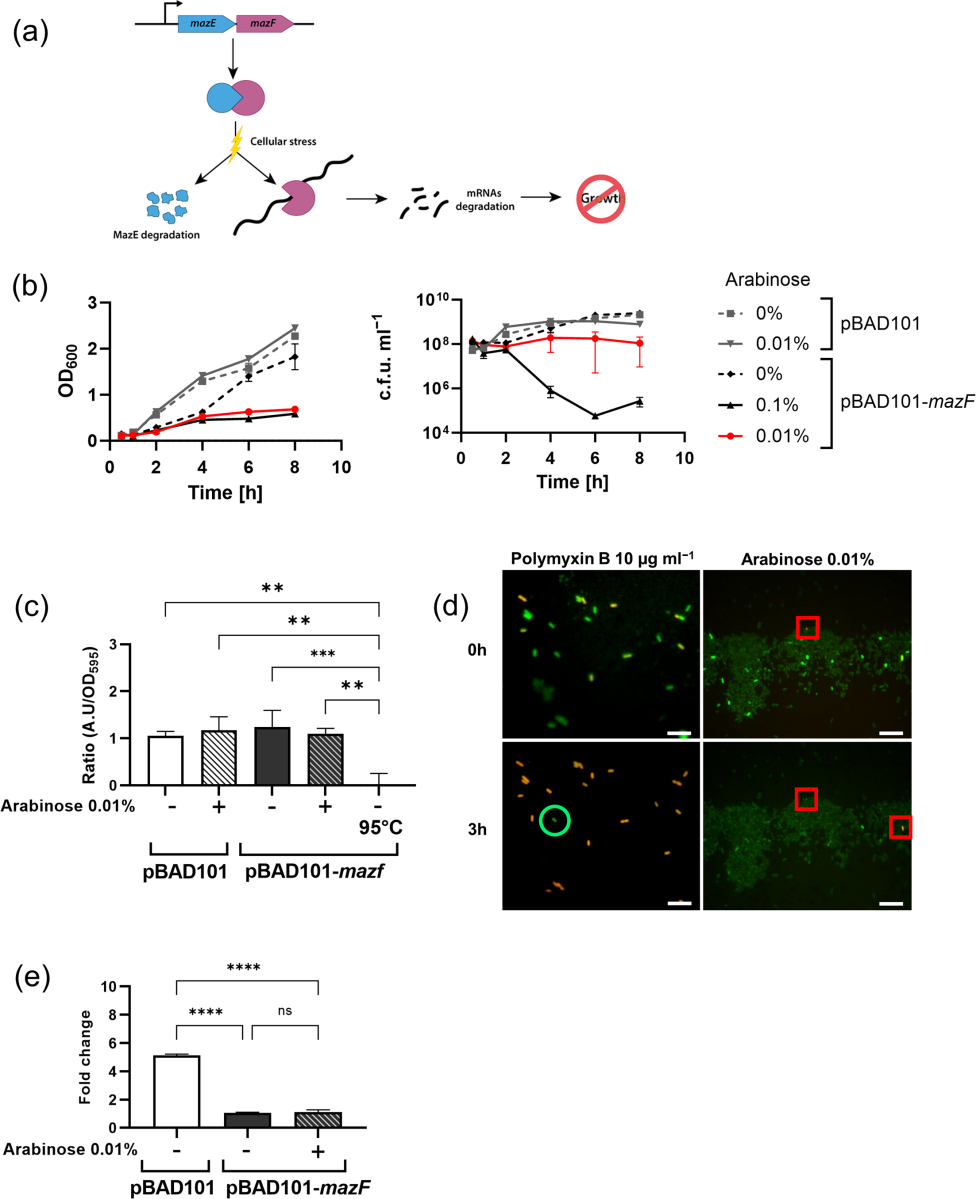
Assessment of bacterial viability and division. (**a**) Mode of action of the MazF–MazE TA system, adapted from Al-Hinai *et al.* [47]. (b) Growth curves of *E. coli* MG1655 pBAD101 or pBAD101-*mazF* in different arabinose concentrations. OD_600_ and c.f.u. ml^−1^ were measured at 30 min, 1 h, 2 h, 4 h, 6 h and 8 h (*n*=3). (c) Resazurin viability assay to measure bacterial viability after 4 h of incubation in the presence or absence of arabinose. Control was done by heat-inactivating *E. coli* MG1655 pBAD101-*mazF* at 95 °C for 5 min. The ratio is the A.U/OD_595_ of the tested conditions divided by the A.U/OD_595_ of *E. coli* MG1655 pBAD101 without arabinose (*n*=3, mean±sd, ns not shown). (**d**) LIVE/DEAD *Bac*Light bacterial viability test to assess single-cell viability of *E. coli* MG1655 pBAD101-*mazF* on the Luria–Bertani (LB) agar pad supplemented with arabinose 0.01% or polymyxin B 10 µg ml^−1^. One image was taken after 0 h and a second after 3 h on the pad. Green cells are alive. Red cells are dead. For the polymyxin B panel, green circle highlights lived cells; for arabinose panels, red squares highlight dead cells. Scale bar=20 µm. (e) Cell division: 1 h culture in the presence or absence of arabinose was plated on an LB agar pad with or without arabinose. One image (630× magnification) was taken at 0 h. A second one was taken 3 h later. Fold change is the ratio of cells at 3 h divided by the number of cells at 0 h (*n*=3, mean±sd is shown) (ns P > 0.05, ** P ≤ 0.01, *** P ≤ 0.001, **** P ≤ 0.0001).

pBAD101‐mazF was a kind gift from S. Ardissone (Institute of Microbiology, Lausanne). The *mazF* gene (from *Waddlia chondrophila*) is controlled by an arabinose‐inducible promoter. This plasmid was developed to study bacteria division in Greub’s group [32]. The mutant strain *E. coli* MG1655 pBAD101-*mazF* expressing the endoribonuclease MazF, the toxin of the well-characterized and highly conserved toxin–antitoxin (TA) system MazF–MazE [33] is used in this study as a slow-growing phenotype. Under normal growth conditions, the toxin (MazF) and the antitoxin (MazE) are co‐expressed and form a stable complex inhibiting the endoribonuclease activity of the toxin (Fig. 1a). However, MazE is degraded under stress conditions and releases MazF to cleave mRNAs and inhibit cell growth [33,34]. In our strain, *mazF* is overexpressed when bacteria are induced with arabinose. Depending on the arabinose concentration, this leads to growth arrest.

pBAD101-*sulA: E. coli* MG1655 *sulA* gene was amplified by PCR using primers *sulA_for_* (5′- AAAAAGCTAGCATGTACACTTCAGGCTATG-3′) and *sulA_rev_* (5′-AAAAAGGTACCTTAATGATACAAATTAGAGTGAA-3′). The PCR fragment was digested with NheI and KpnI enzymes and then ligated into pBAD101, restricted with NheI and KpnI sites. The *SulA* gene is also under the control of an arabinose-inducible promoter. To obtain growing but non‐dividing bacteria, we transformed MG1655 pBAD101‐*sulA* under the control of the *araC* promoter. *SulA* is implicated in the SOS response triggered by DNA damage. It directly interacts with FtsZ and inhibits FtsZ polymerization, resulting in the absence of the Z‐ring [35,36]. The phenotype is characterized by a lack of septation, resulting in cell separation failure and producing elongated bacteria(Fig. 4a).

Plasmids were amplified in TOP10 *E. coli*, and the presence of the *mazF* or *sulA* gene was assessed by PCR using *pBAD_for_* (5′-CTACCTGACGCTTTTTATCGCAA-3′) and *pBAD_rev_* (5′-GCGTTCTGATTTAATCTGTATCAGG-3′) primers. Positive plasmids were used to transform *E. coli* MG1655.

*E. coli* MG1655 pBAD101, *E. coli* MG1655 pBAD101-*mazF* and *E. coli* MG1655 pBAD101-*sulA* were routinely grown at 37 °C with 200 r.p.m. shaking in Luria–Bertani (LB) broth supplemented with streptomycin 100 µg ml^−1^. When required, gene expression was induced by arabinose (Sigma-Aldrich, Saint Louis, MO, USA).

### Growth and kill curves

Overnight cultures were diluted to an optical density of 0.1 at 600 nm (OD_600_) in 3 ml of medium supplemented or not with arabinose or antibiotics of interest. Antibiotics used were ampicillin (AppliChem, Darmstadt, Germany) and polymyxin B (Sigma-Aldrich). Absorbance at 600 nm was measured at different time points (0.5, 1, 2, 4, 6 and 8 h), and the number of c.f.u. was assessed. Briefly, the culture was serially diluted and plated on LB agar (LB supplemented with 15% agarose) with 100 µg ml^−1^ streptomycin. Dilution plates where single colonies (10–50) were easily countable were used to calculate the c.f.u. ml^−1^.

### Measurement of bacterial viability

To assess bacterial viability under arabinose induction, overnight cultures were diluted to OD_600_=0.1 in 96-well plates. Plates were then incubated for 4 h at 37 °C, and absorbance was measured at 590 nm. As a control, a 4-h bacterial culture was heated for 5 min at 95 °C. Then, bacteria were incubated with 10 µg ml^−1^ resazurin (Sigma-Aldrich), and plates were incubated for 15 min at 37 °C. Fluorescence was measured with a FLUOstar Omega Microplate Reader (BMG LABTECH, Ortenberg, Germany), with an excitation/emission wavelength of 540/580 nm. Fluorescence was standardized according to the last absorbance measure.

### Single-cell division and live–dead viability test

To assess cell division and single-cell viability, overnight cultures were diluted to an OD_600_=0.1 and incubated for 1 h at 37 °C with 200 r.p.m. shaking in the presence or absence of arabinose. Afterwards, 5 µl bacteria were plated on an LB agar pad supplemented or not with arabinose or 10 µg ml^−1^ of polymyxin B. The liquid drop was air-dried for 5–10 min before being covered with a cover slip. Finally, two pictures (at 0 and 3 h) were taken at 630× magnification with the Axioplan 2 microscope (Zeiss, Oberkochen, Germany). A cover slip was kept under the lens until the last picture was taken to keep the same field during the entire experiment. For the single-cell viability test, bacteria were stained according to the manufacturer’s instructions with the LIVE/DEAD *Bac*Light Bacterial Viability Kit (Thermo Fisher) before being plated on the pad.

### pBAD101-*sulA* phenotype

Overnight cultures were diluted to an OD_600_=0.1 and incubated for 1 h at 37 °C with 200 r.p.m. shaking in the presence or absence of arabinose. Ten microlitres of bacteria were taken at 0 and 4 h and plated on the cover slip. Finally, pictures were taken at magnificent 630× with the Axioplan 2 microscope (Zeiss).

### MIC measurement

Overnight cultures were diluted to an OD_600_=0.1 in 96-well plates containing serial dilutions of antibiotics (polymyxin B 0.0625–32 µg ml^−1^ ; ampicillin 0.25–256 µg ml^−1^). Plates were incubated statically for 24 h at 37 °C, and absorbance was measured at 590 nm. The lowest concentration at which no turbidity was observed was determined as the MIC.

### Nanomotion

Nanomotion technology was initially developed at Ecole Polytechnique Federal Lausanne by the group of Giovanni Dietler and Sandor Kasas, where they successfully detected and measured the nanomotion of living organisms, as bacteria [24]. One of the potential applications of this technology is to evaluate the viability of micro-organisms and their responses when exposed to killing agents such as antibiotics. In collaboration with Greub’s group, this new technology was tested on real blood culture samples [26].

For nanomotion experiments, we used the Phenotech device and corresponding sensors with cantilevers (Resistell AG, Muttenz, Switzerland). Sensors were preliminary functionalized with poly-d-lysine 0.01% (Sigma-Aldrich) for 20 min to ensure a firm attachment of bacteria and were subsequently washed and dried.

Overnight cultures were diluted to an OD_600_=0.1 in 3 ml of LB+streptomycin 100 µg ml^−1^ and incubated for 1 h at 37 °C with 200 r.p.m. shaking. Bacteria were pelleted by centrifugation at 5000 ***g*** for 3 min and resuspended in 200 µl PBS. Then, the sensor was incubated 5 min in the 200 µl PBS drop to attach bacteria to the cantilever. Unattached bacteria were removed by gently washing with PBS.

The sensor with bacteria attached to the cantilever was introduced in the measurement chamber containing LB+streptomycin 100 µg ml^−1^ supplemented with arabinose. For experiments with antibiotics, the drug was added directly in the medium after 1 h of recording. Added antibiotics were mixed by pipetting two to three times up and down.

### Nanomotion analysis and feature selection

The nanomotion signal is collected at a sampling frequency of 60 kHz and represented as a time series and is divided into 10 s time frames. Linear trends were removed, and variance was calculated for each time frame. Noise required smoothing using a 1-min running median. Variance plots served as the main tool for visual inspection in this study and sufficed to calculate the slope, i.e. condense the curve information into a single numerical value, the so-called feature ‘slope’. The slope of the nanomotion recordings was calculated using the following equation:

$x=C^{kt}\;\text{or log}(x)=\text{log}(C)+kt,$

where *t* is the time, *k* is the slope and *C* is the intercept.

Using the variance or other simple statistical features calculated from the nanomotion signal does not exhaust the information in the signal. Since the signal is extremely dense, i.e. a 60 kHz sample frequency, automated computing power is needed to calculate a pre‐defined set of mathematical features from the curve or parts of it. Supervised machine learning can extract and select these features to train a classification model that discriminates experiments under different conditions or strains, for instance, fast‐growing versus slow‐growing bacteria.

Extracting features from the signal is necessary to apply supervised machine learning techniques. This process involves the estimation of the positions and amplitudes of characteristic shapes within the power spectrum. To mitigate the impact of noise, these estimated values are subjected to statistical aggregation within fixed time windows of 20 min.

The base features are structured in Table 2.

**Table 2.** Structure of base features: ‘Bac_S3_161_181_median’

Feature element	Description	Example
Experimental phase	General experimental phase (medium only, with bacteria and with drugs added)	Bac
Signal estimator on power spectrum	Evaluation metric, such as the slope of a linear fit within a specific frequency range	S3
Time window	Start and end time of statistical aggregation window (in minutes)	161_181
Statistical aggregation function	Used to aggregate the values from the signal estimator to mitigate noisy observations	Median

As these base features have only a limited time horizon, in this case, the 20 min from the aggregation window, it is beneficial to combine them with composition features in order to increase the said time horizon. These composition features can be derived from the base features by employing mathematical operations such as division or, as illustrated in the provided example, subtraction. By merging information from two different time windows, more descriptive features can be formed.

For instance, considering the growth feature, it is structured as follows: ‘Bac_S3_161_181_median_minus_Bac_S1_101_121_median’. In this case, it delineates a relationship between two base features (S1: y-intersect of a linear fit to the log-log transformed power spectrum at a frequency interval of 5–10 Hz and S3: y-intersect of a linear fit to the log-log transformed power spectrum at a frequency interval of 10–50 Hz) from two distinct time windows (161–181 min and 101–121 min). Therefore, it extracts much more specific information of interest from the signal than by using simple statistics or also using only the aforementioned base features.

A feature selection algorithm reduces the resulting high‐dimensional feature space to a few features. In this case, a wrapper method called forward selection is applied [37], which selects every feature in the model and selects the one with the highest accuracy estimated by the *k*‐fold cross‐validation method. In addition, the model fitting algorithm has been used to reduce overfitting due to the small sample size apart from the *k*‐fold cross‐validation L2‐regularizer [38].

### Data analysis

Prism 9 for Windows (GraphPad Software) was used for statistical analyses. Ordinary one-way ANOVA with Bonferroni correction for multi-testing was used with ns *P*>0.05, *P≤0.05, **P≤0.01, ***P≤0.001, ****P≤0.0001.

## Results

### Nanomotion to differentiate growing and slow-growing bacteria

#### Viability of slow-growing bacteria

The slow‐growing mutant used in these experiments is *E. coli* MG1655 pBAD101*‐mazF*. The *mazF* endoribonuclease is overexpressed when bacteria are induced with arabinose. Depending on the arabinose concentration, this leads to growth arrest.

Firstly, we determined which arabinose concentrations were needed to stop or slow down bacterial growth without killing the cell. Bacterial growth was measured at OD_600_ and viability by c.f.u. ml^−1^ (Fig. 1b). During the first 4 h of culture, the growth rates of MG1655 pBAD101-*mazF* with 0%, 0.01% and 0.1% arabinose were 2-, 2.44- and 2.85-fold lower, respectively, than that of the WT with the empty vector control without arabinose. A deferred growth arrest was observed under *mazF*-inducing conditions in the presence of 0.01% or 0.1% arabinose after 4 h. During the first 2 h of culture, bacterial viability was similar in all conditions tested for both strains. The exposure to 0.1% arabinose eventually induced cell death for bacteria carrying pBAD101-*mazF*, while an arabinose concentration of 0.01% reduced the growth without significantly affecting viability. Thus, this concentration was chosen for further experiments (despite high variability in c.f.u. ml^−1^). To ensure that experiments were performed with highly viable bacteria, we decided to reduce the time window of further experiments to 4 h.

A resazurin–resorufin redox assay was used to measure the metabolic activity of the bacterial cells, which was then used to deduce their viability (Fig. 1c). Independent of the presence or induction of *mazF* (for 4 h) at 0.01% arabinose, the tested conditions showed similar resazurin–resorufin ratios. A 4-h induction of 0.01% arabinose did not affect the viability of MG1655 pBAD101-*mazF*, which was further corroborated by a single-cell-based LIVE/DEAD *Bac*Light assay (Fig. 1d). Bacteria were first incubated for 1 h in liquid culture supplemented with arabinose, stained with LIVE/DEAD *Bac*Light assay and then plated on an LB agar pad supplemented with arabinose. After 3 h of induction with 0.01% arabinose on an LB agar pad, the *mazF*-expressing bacteria stained green-fluorescent, indicative of an intact membrane. While in the control experiment, it stained red and thus lost membrane integrity on LB agar pads within 3 h of incubation when it contained 10 µg ml^−1^ of polymyxin B (MIC 2 µg ml^−1^), a fast-killing drug. It confirms the low impact of arabinose induction on the viability of MG1655 pBAD101-*mazF* during the first few hours of incubation.

In a second microscopy-based single-cell assay, we assessed the average number of cell divisions within 3 h. Practically, bacteria were spread on an LB agar pad under induced and uninduced conditions (Fig. 1e). MG1655 pBAD101 reached a fold change of 5.1, while MG1655 pBAD101-*mazF* was not dividing, regardless of arabinose induction – most likely due to a certain level of leakage in the *araC* promoter, leading to low but sufficient levels of MazF even in non-induced conditions. In contrast to the slow growth observed in liquid culture (Fig. 1b), no division was observed on the LB pad. The limited oxygen availability and lower ambient temperature (room temperature) during the incubation on the LB agar pad are very likely accountable for this difference compared with shaking liquid culture at 37 °C.

#### Nanomotion of slow-growing bacteria

It has been demonstrated that living bacteria emit more vibrations than drug-inactivated bacteria [24,26, 27, 29,31] (Fig. 3a). However, signals of exponentially growing or slow-growing bacteria have never been compared. To that end, we attached MG1655 pBAD101 or MG1655 pBAD101-*mazF* on cantilevers (Fig. 3b) and measured their nanomotion in the presence or absence of arabinose for 3 h (Fig. 3c). The deflections are given as the variance over 10 s. The higher the variance, the higher the amplitude of those deflections. Independent of arabinose induction, MG1655 pBAD101 showed a higher variance than MG1655 pBAD101-*mazF* (Fig. 3c). In addition, by looking at the slope of the variance of each replicate (Fig. 3d), we observed that growing cells of MG1655 pBAD101 tend to have a steeper variance slope than the slow-growing cells of MG1655 pBAD101-*mazF*, even though differences were not statistically significant. Moreover, in the presence of arabinose, the strain carrying pBAD101-*mazF* displays a slope close to 0.

**Fig. 2.**
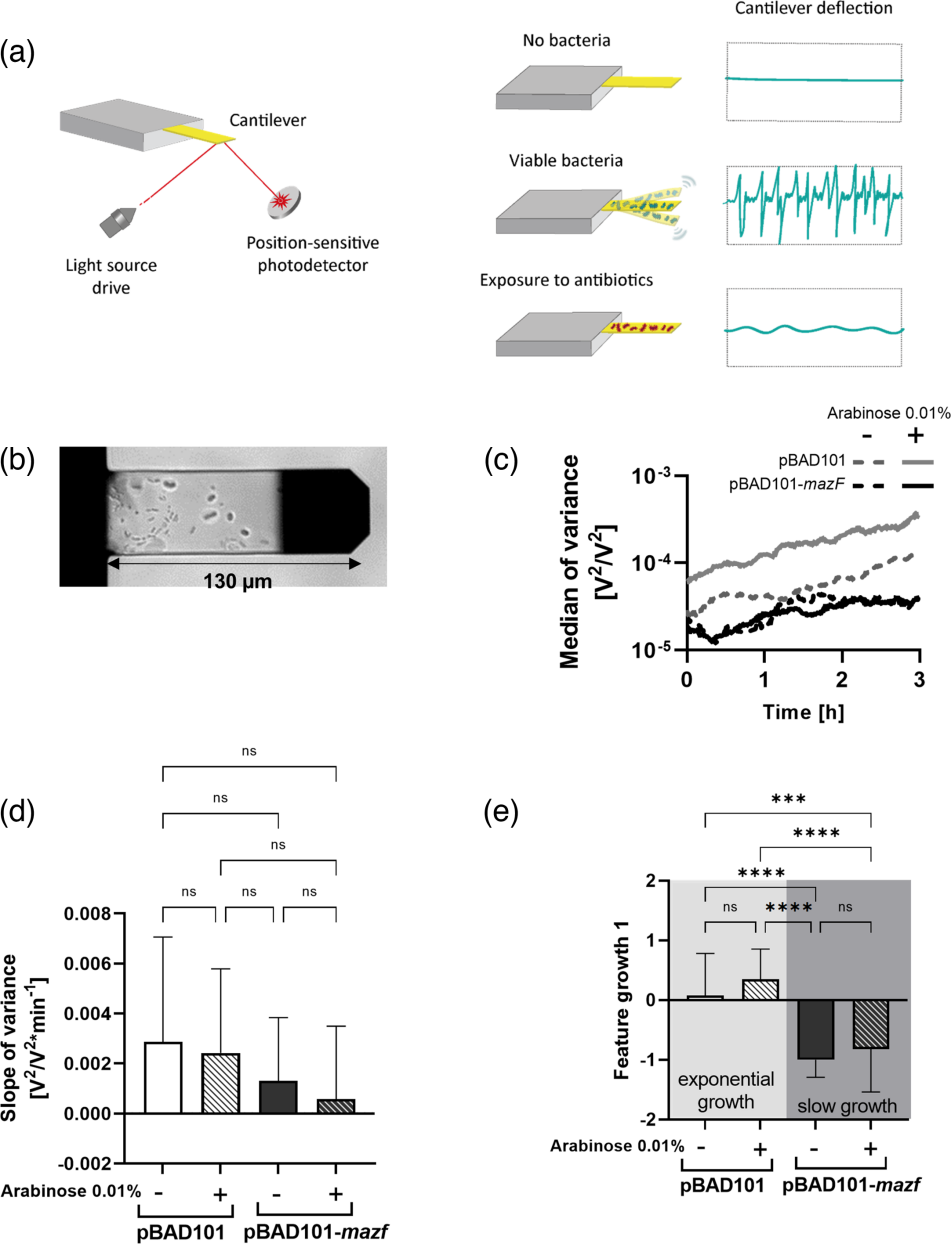
Nanomotion of slow-growing cells. (**a**) Left panel: cantilever deflection is recorded by fluctuations of the light beam on the position-sensitive photodetector. Right panel: fluctuations measured on a nanomotion sensor (functionalized with a linking agent) correspond to environmental noise and are low. After the attachment of living bacteria, fluctuations are high and are driven by the metabolically active bacteria. After exposure to killing agents such as antibiotics, fluctuations return to a low level – adapted from Stupar *et al.* [26]. (**b**) Cantilever with *E. coli* MG1655 attached to it. The black tip is the mirror on which the light source is reflected to reach the position-sensitive photodetector. (**c**) Nanomotion of *E. coli* MG1655 carrying pBAD101 or pBAD101-*mazF* in the presence or absence of arabinose. Mean, *n*=18. (**d**) Slope of variance from (c) (*n*=18). (e) The best feature is allowing for the differentiation of slow-growing and growing cells (*n*=18) (ns P > 0.05, *** P ≤ 0.001, **** P ≤ 0.0001).

**Fig. 3.**
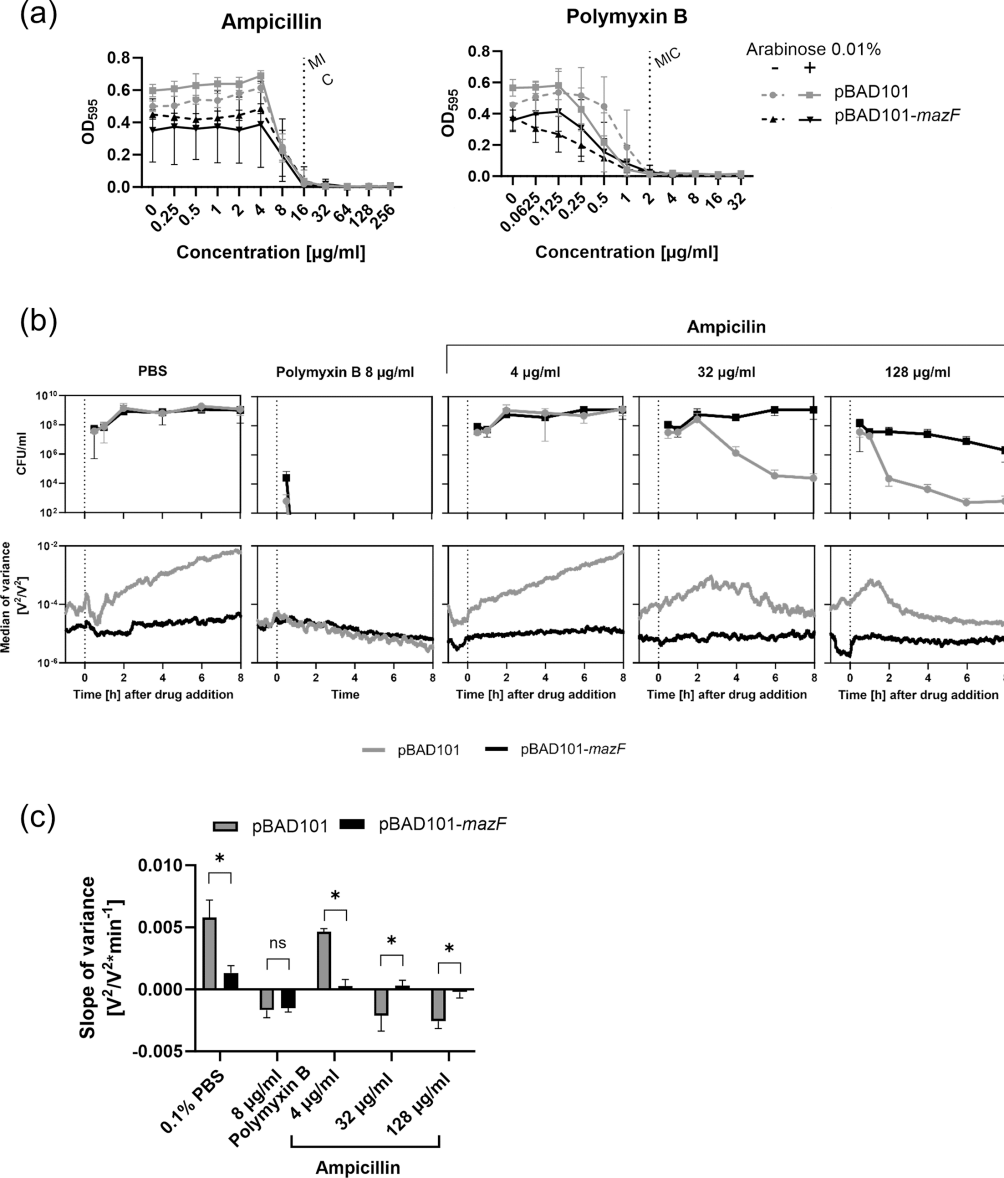
Nanomotion in the presence of antibiotics: (a) Determination of MIC for ampicillin and polymyxin B. *n*=3, mean±sd. (**b**) Kill curves (upper panel) and nanomotion (lower panel) of *E. coli* MG1655 carrying pBAD101 (grey) or pBAD101-*mazF* (black). Nanomotion: *n*=4, Mean±sem, Kill curve: *n*=3, mean±sd. (**c**) Slope of variance for *E. coli* MG1655 carrying pBAD101 (grey) or pBAD101-*mazF* (black). Mean±sd. Mann–Whitney test was used. (ns P > 0.05, * P ≤ 0.05).

We used 18 experiments to train a model based on a single feature from the nanomotion signal that could differentiate growing and slow-growing cells (Fig. 3d and e). This feature, named ‘growth 1’, could distinguish MG1655 pBAD101 and pBAD101-*mazF*, respectively (Fig. 3e). However, it could not differentiate induced versus uninduced (Fig. 3e).

The feature ‘growth 1’ (Bac_S3_161_181_median_minus_Bac_S1_101_121_median) is computed by taking the difference of the signal in two different frequency ranges (10–50 Hz and 5–10 Hz) during two different time windows (160–180 min and 100–120 min, respectively). This means that ‘growth 1’ captures the trend in these frequency bands over time, which implies that the underlying process is changing.

### Nanomotion as an indicator of tolerance

Tolerance, the ability of bacteria to survive a transient exposure to antibiotics, is typically observed for non- or slow-growing bacteria [4,7, 9,12, 39, 40]. Therefore, we could establish that the slow-grower MG1655 pBAD101-*mazF* comprised differences in the nanomotion signal compared with the fast-grower MG1655 pBAD101. Because of the different growth phenotypes, a different tolerance toward antibiotic-targeting cell growth can be expected. Thus, ampicillin was selected, a β-lactam inhibiting the cell wall peptidoglycan synthesis [41]. As a control, we chose polymyxin B, which disrupts the bacterial membranes in a growth-independent manner [42].

**Fig. 4.**
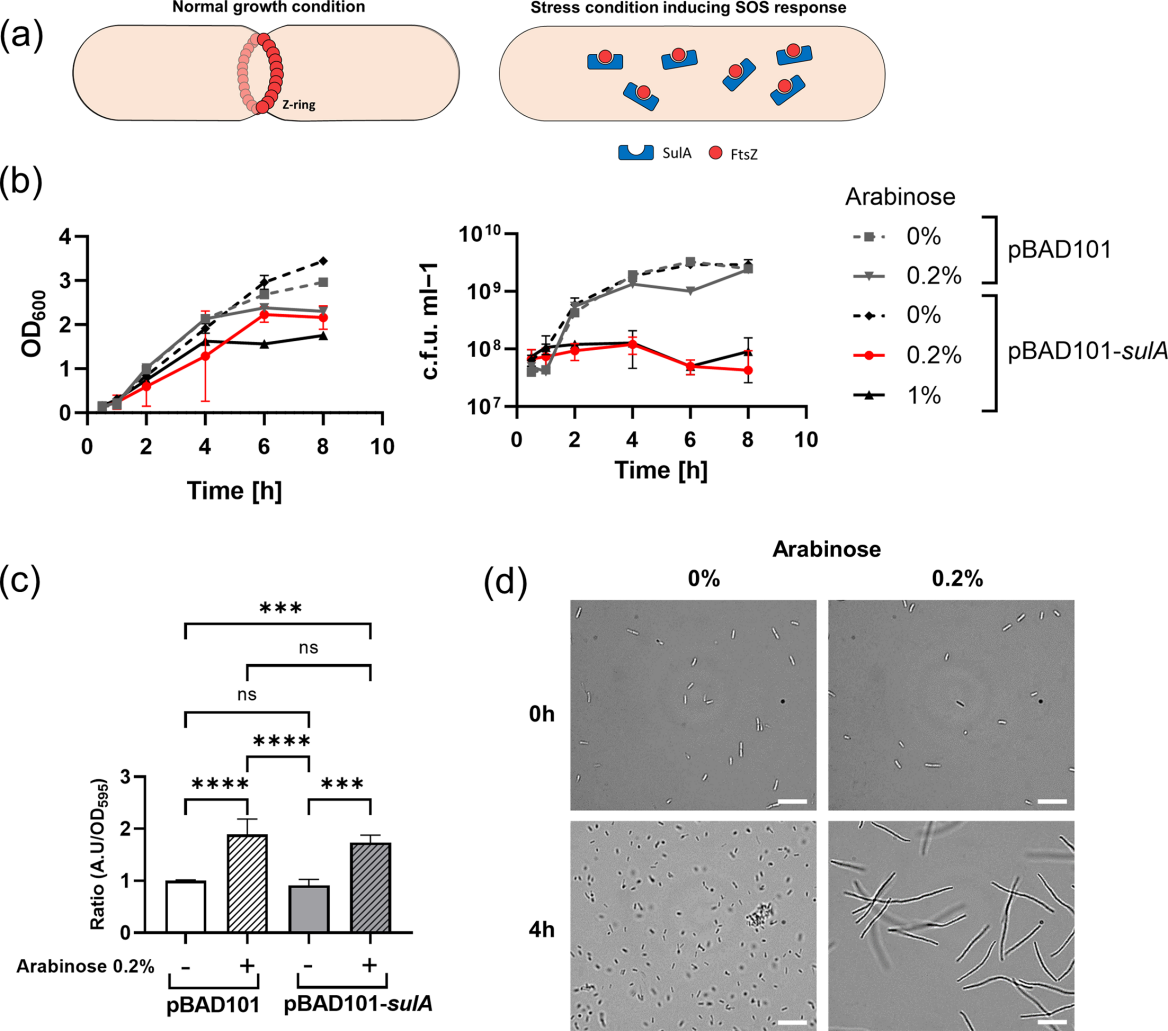
Overexpression of *SulA* blocks the formation of the Z-ring and results in an elongated phenotype. (**a**) Mode of action of *SulA*. Under stress conditions inducing the SOS response, *SulA* is expressed and binds the monomer of FtsZ, preventing the formation of the Z-ring. (**b**) Growth curves of *E. coli* MG1655 carrying pBAD101 or pBAD101-*sulA* in different arabinose concentrations. OD_600_ and c.f.u. ml^−1^ were measured at 30 min, 1 h, 2 h, 4 h, 6 h and 8 h (*n*=3). (c) Resazurin viability assay to measure bacterial viability after 4 h of incubation in the presence or absence of arabinose. The ratio between A.U/OD_595_ tested conditions and the A.U/OD_595_ of *E. coli* MG1655 pBAD101 was calculated (*n*=3, mean±sd) (ns P > 0.05, *** P ≤ 0.001, **** P ≤ 0.0001). (**d**) Cell phenotype after induction. Scale bar=20 µm.

In the first step, we determined the MIC for both drugs in a broth microdilution assay in the presence or absence of arabinose. For both MG1655 derivatives, we detected an MIC of 16 µg ml^−1^ for ampicillin and 2 µg ml^−1^ for polymyxin B independent of arabinose (Fig. 4a). Because of the suspected *araC* promoter leakage, we abandoned using arabinose in the following nanomotion experiments with sub-inhibitory and inhibitory antibiotic concentrations [ampicillin: 4 µg ml^−1^ (=MIC/4), 32 µg ml^−1^ (=2× MIC) and 128 µg ml^−1^ (=8× MIC); polymyxin B: 8 µg ml^−1^ (=2× MIC)]. We used PBS instead of the two drugs as a negative control. To that end, bacteria were cultured in a liquid culture medium for 1 h before being attached to the cantilever, and the nanomotion measurement commenced. After 1 h of nanomotion recording, the antibiotic was added directly to the measurement chamber, and the recording continued for 8 h (Fig. 4b). In parallel, the kill rate was assessed by counting c.f.u. ml^-1^ (Fig. 4b).

We considered the exponential slope (*k*) of the variance an indicator of bacterial viability. Therefore, we suspected a negative slope for dying cells, while tolerant cells should depict an increase or at least stagnation in the variance over time. Indeed, we observed a significant positive trend of the variance over time for MG1655 pBAD101 (*k*=5.78×10^−03^) and a four times lower rate for MG1655 pBAD101-*mazF* (*k*=1.23×10^−03^) in the PBS control experiments (Fig. 4c). This significant slope difference between the two strains could be attributed to the extended recording time of 8 h (Fig. 4c), which contrasts with the previous experiment (Fig. 3d). Furthermore, the number of c.f.u. ml^−1^ was similarly increasing with both strains during the first 2 h of incubation with PBS (Fig. 4b), suggesting that the differences in variance are not due to differences in the biomass on the cantilever.

We still observed a positive slope when bacteria were incubated with sub-inhibitory concentrations of ampicillin (4 µg ml^−1^, 0.25× MIC), although the slopes of slow-growing bacteria were lower than in the PBS control (pBAD101: *k*=4.64×10^−03^; pBAD101-*mazF*: *k*=2.54×10^−04^). By increasing the antibiotic concentration to 64 µg ml^−1^, equivalent to twice the MIC (32 µg ml^−1^), we observed that the variance of MG1655 pBAD101 and c.f.u. ml^−1^ decreased after 2 h of the addition of ampicillin (*k*=−2.13×10^−03^). With 128 µg ml^−1^ ampicillin (8× MIC), the variance and c.f.u. ml^−1^ drop were simultaneously observed after 1 h incubation with ampicillin (*k*=−2.57×10^−03^). The slope was 1.21-fold steeper than that obtained at 32 µg ml^−1^. Moreover, the curve increased between 1 and 3 h steeply after ampicillin addition. It stabilized, contrary to 32 µg ml^−1^, where the curve decreased linearly after 2 h.

Contrary to the fast-grower MG1655 pBAD101, when the slow-grower MG1655 pBAD101-*mazF* was treated with 32 µg ml^−1^ ampicillin, the slope of the variance did not change compared to sub-inhibitory concentrations of ampicillin (*k*_32µg_=2.93×10^−04^ versus *k*_4µg_=2.54×10^−04^). This was in line with the observation that the c.f.u. ml^−1^ did not alter over time (Fig. 4b). However, treating the slow-growing strain with 128 µg ml^−1^ ampicillin resulted in a slight decrease in c.f.u. ml^−1^ after 4 h of incubation. This was reflected in the slope of the variance that assumed a negative value suggesting a decreased viability (*k*_128µg_=−2.32×10^−04^) (Fig. 4c). Finally, for inhibitory concentrations of polymyxin B (four times the MIC), we observed similar results with fast-growing and slow-growing strains, as expected, given that its mode of action is independent of growth (Fig. 4b and c). For both strains, the number of c.f.u. ml^−1^ dropped at the same time, and the slopes of the variance assumed comparable negative values (*k*_pBAD101_=−1.67×10^−03^; *k*_pBAD101-*mazF*_ =−1.51×10^−03^).

### Nanomotion to characterize growing but not dividing bacteria

By measuring the nanomotions of bacteria exposed to an antibiotic, it is possible to differentiate bacteria that get killed (susceptible) from those that can proliferate (resistant) [24,26, 27, 29,31]. However, susceptible bacteria can undergo morphological changes before getting killed. For example, in the case of cephalosporins, susceptible bacteria can elongate while losing the ability to divide [43]. Therefore, we wondered if nanomotion could distinguish elongating bacteria from dividing cells, as observed in microscopic techniques.

The growing but not dividing bacterial strain MG1655 pBAD101-*sulA* is under the control of the *araC* promoter, inducible using arabinose. To determine the arabinose concentration needed to stop cell division without reducing biomass production or killing the cell, we measured the OD_600_ and c.f.u. ml^−1^ of MG1655 pBAD101 and MG1655 pBAD101-*sulA* (Fig. 2b). At 0.2% arabinose, we observed an increase in OD_600_ but no increase in c.f.u.s. Hence, it was chosen from several different concentrations. Higher concentrations resulted in a decrease in biomass production of MG1655 pBAD101-*sulA*, while lower concentrations were not sufficient to stop bacterial division. We tested further the viability of both strains in the presence or absence of arabinose induction using resazurin. We observed no difference between MG1655 pBAD101 and MG165 pBAD101-*sulA*, but a twofold increased viability in the presence of arabinose (Fig. 2c), most likely explained by higher metabolism due to the additional nutrient source arabinose. Finally, we confirmed the expression of *sulA* by looking at cell morphology microscopically after 4-h induction (Fig. 2d). MG1655 pBAD101-*sulA* cells were elongated and were up to sevenfold longer than non-induced cells.

**Fig. 5.**
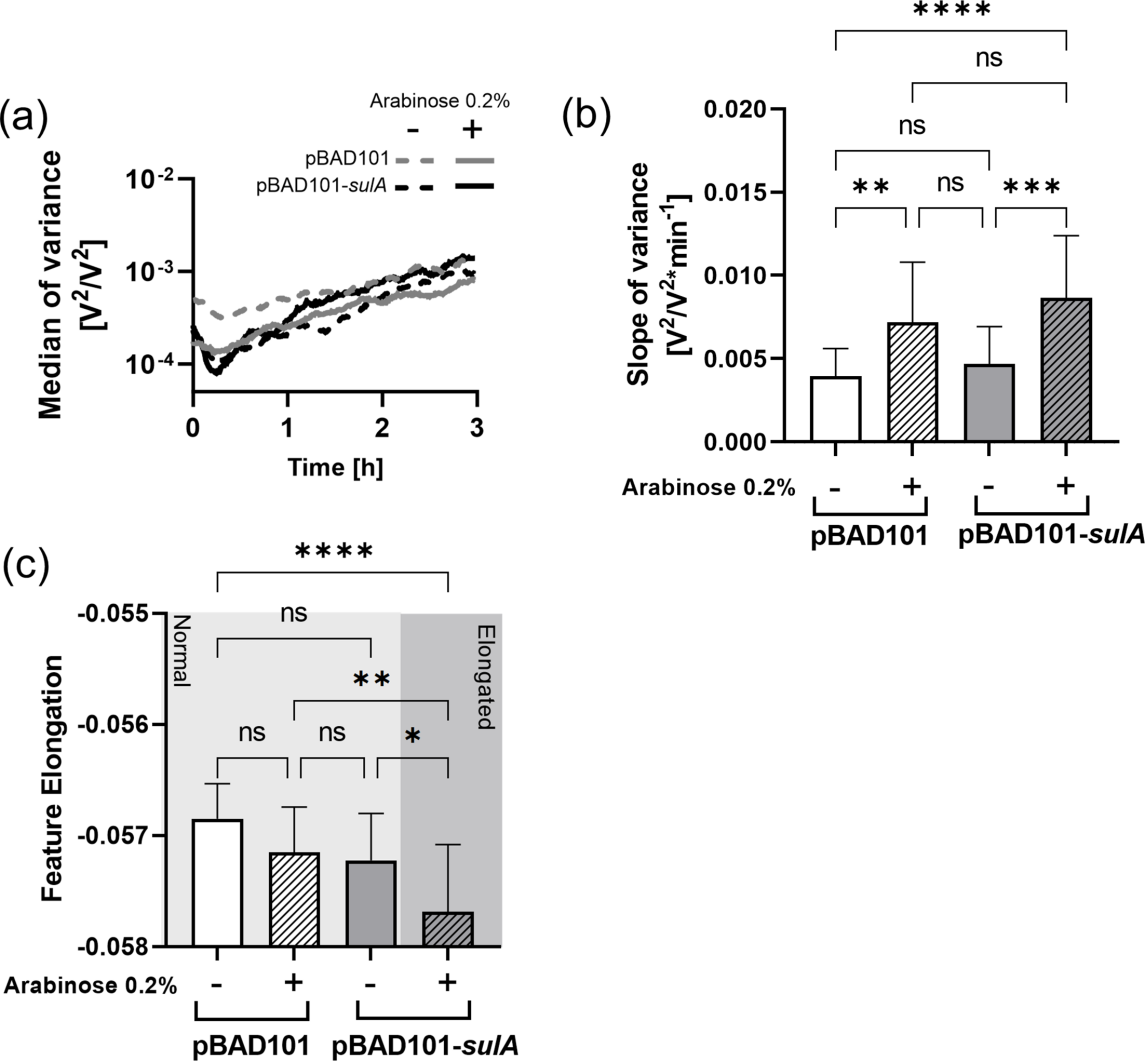
Nanomotion of growing but non-dividing bacteria. (a) Nanomotion of *E. coli* MG1655 carrying pBAD101 and pBAD101-*sulA* in the presence or absence of arabinose. Mean, *n*=18. (**b**) Slope of cantilever variance from (a) (*n*=18). (c) The best feature allowing differentiation of growing cells from growing but non-dividing (elongated) ones (*n*=18) (ns P > 0.05, * P ≤ 0.05, ** P ≤ 0.01, *** P ≤ 0.001, **** P ≤ 0.0001).

We compared the elongating MG1655 pBAD101-*sulA* nanomotion with the fast-growing MG1655 pBAD101 in the presence or absence of arabinose (Fig. 5a). The variance increased over time for both strains and conditions, and no significant slope difference was observed between uninduced strains (Fig. 5b). However, when arabinose was added, the variance slope was significantly higher in both strains, reflecting the results previously obtained by the resazurin assay of increased metabolism due to the surplus of nutrients (Figs2c  5b).

Finally, we used this set of nanomotion recordings to identify by machine learning putative features that could differentiate dividing growing cells from those growing but non-dividing. The feature named ‘elongation 1’ (Bac_S0-rise-rp_100_120_q95_over_Bac_S1-rise-rp_60_80_q95) could distinguish strains with exponential growth (pBAD101 with or without arabinose and pBAD101-*sulA* without induction) from elongated bacteria (pBAD101-*sulA*+0.2% arabinose) (Fig. 5c). The feature ‘elongation 1’ characterizes the noise content in the high-frequency part (6000–9000 Hz) of the power density spectrum of the signal. It is calculated as the ratio between two-time windows at 100–120 min and 60–80 min, respectively. This feature explains how the signal shape in these frequency ranges changes over time.

## Discussion

Nanomotion technology has been used to rapidly evaluate the antibiotic susceptibility of several bacteria under standard laboratory culture conditions [24,26, 27, 29,31]. For the first time, we showed vibration measurements of an *E. coli* growing two times slower than the WT and which is tolerant to β-lactams by using the conserved MazEF system [33,34]. Based on information in the variance of 3 h of nanomotion measurements, exponentially growing and slow-growing bacteria could be distinguished using a feature determined by machine learning. Importantly, nanomotions or bacterial vibrations are widely independent of growth and depend on the cells’ metabolic activity. However, the variance increase over time might be attributed to growth and is further corroborated by even steeper increases upon the addition of the metabolite arabinose to the media [44,45]. Furthermore, the variance increase over time in culture media was shown before for *B. pertussis* using an exponential fit [29]. Consequently, slow-growing bacteria showed a lesser increase.

With a sampling frequency of 60 kHz, the signal is complex, spanning a wide range of frequencies. However, the variance changes towards antibiotic stress are best understood [24,26, 27, 29,31]. The feature ‘growth 1’ was selected from a large set of features describing signal changes over time. Applying machine learning methods to discriminate best between both phenotypes was found in the ratio of the low-frequency range of the power density spectrum between 160–180 min and 100–120 min (Fig. 3e). As it relates two intervals in the signal to each other, this feature can be understood as a refinement of the slope (Fig. 3d). It shows that in our experimental setting, both phenotypes differ in their nanomotion behaviour. However, it does not allow us to conclude about biological underlying mechanisms. Similar conclusions can be drawn for the second feature (elongation) selected by machine learning, i.e. that a specific signal may be empirically detected. The information for elongation was drawn from much higher frequencies of the spectrum (6000–9000 Hz), relating the time interval at 100–120 min to the one at 60–80 min. Future works will show if these features can be further generalized and related to a specific underlying biological mechanism. It still remains to be determined why both features derive from different power spectrum frequencies. In future works, the elongation triggered by SulA must also be compared with elongation induced by antibiotics, such as fluoroquinolones or β-lactams.

The significant finding in this work is the real-time assessment of antibiotic tolerance using nanomotion technology, which reduces the time to result by approximately 18–24 h compared with time-kill curves. With standard methods like time-kill curves, tolerant patterns are observable within the first 8 h. By focusing on this same time window, we successfully measured nanomotion deflection over 8 h.

Standard methods, such as time-kill curves, involve a significant workload and have a severe blind spot between actual bacterial death and the inability to form a colony, making them impractical for routine use. The minimum bactericidal concentration (MBC)/MIC ratio has also been proposed as a measure of bacterial tolerance [46]. The MBC represents the lowest concentration of an antibiotic required to kill ≥99.9% of cells in a bacterial culture, typically determined after 24 h of incubation. However, this method is more time-consuming compared with the nanomotion methods we propose here.

The overexpression of the toxin *mazF* is suitable for studying tolerance as it confirms that ampicillin requires growth for efficacy as determined by the classical method and the novel nanomotion technology [11,14,17]. From previous works, we knew that β-lactams would decrease the variance in the case of susceptibility [24,26, 27, 29,31]. Furthermore, we showed in this work by testing a susceptible strain that the decrease in variance is dose-dependent. However, when a tolerant strain expressing *mazF* was tested, even at very high inhibitory concentrations, we did not see the variance decrease supported by the classical c.f.u. kill-curves assay (Fig. 4b). Improving our understanding of tolerance mechanisms will allow us to address recurring and chronic infections. However, further experiments with other mechanisms triggering tolerance are needed, such as starvation, immune factors or antibacterial drugs. In addition, different bacterial strains, especially clinical isolates, are now required to increase the evaluation of this method (and its robustness by a better machine learning algorithm) before having a method ready to get applied in the diagnostic setting to detect antibiotic tolerance.

## Abbreviations

AFM: atomic force microscopy
LB: Luria–Bertani broth
MBC: minimum bactericidal concentration
MDK: minimum duration of killing
TA: toxin–antitoxin
